# Structure of early signs of affective pathology in adolescents

**DOI:** 10.1192/j.eurpsy.2021.1678

**Published:** 2021-08-13

**Authors:** N. Osipova, L. Bardenshteyn, N. Beglyankin, G. Aleshkina, M. Dmitriev

**Affiliations:** 1 Department Of Psychiatry And Narcology, A. I. Yevdokimov Moscow State University of Medicine and Dentistry, Moscow, Russian Federation; 2 Department Of Psychiatry And Narcology, A. I. Evdokimov Moscow State University of Medicine and Dentistry, Moscow, Russian Federation; 3 Department Of Physics, Mathematics And Medical Informatics, Smolensk State Medical University, Smolensk, Russian Federation

**Keywords:** adolescents, early diagnosis, affective pathology

## Abstract

**Introduction:**

Studies in adults with bipolar disorder (BD), shows that in 25% of cases first affective episode occurs under the age of 13 and in 63-69% under the age of 19. The most difficult problem is the early identification of BD, which starts in adolescence as a result of polymorphism of clinical symptoms, their syndromic incompleteness.

**Objectives:**

Study of the structure of adolescents affective disorders on primary appointment in outpatient psychiatric unit.

**Methods:**

Content analysis, sampling method, statistical method. 120 disease histories of adolescents who first applied for outpatient psychiatric unit in 2019 were used. 93 (77.5%) of them were girls and 27 (22.5%) of them were boys. The average age was 17 years.

**Results:**

In the structure of initial diagnoses, according to ICD-10, mood disorders [F30-F39] - 56.0% prevailed. [F40-F49] - 25%, [F00-F09] - 6.6%, [F20-F29] - 6.6%, [F50-F59] – 4,2%, [F90-F99] – 1,6% were less likely. Structure of complaints of adolescents and their parents on primary appointment for specialized psychiatric care is shown in Table 1 (p<0,05).

**Figure:**
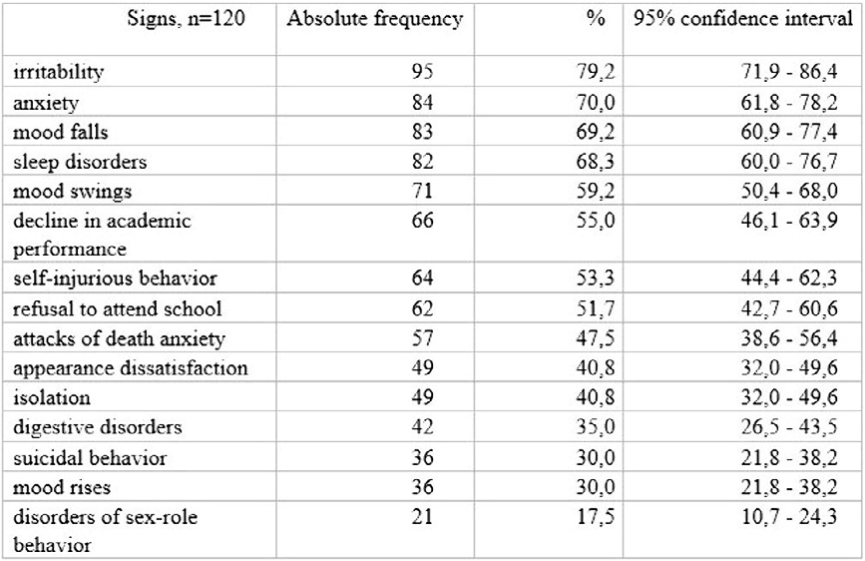

**Conclusions:**

Initial signs of emotional disorders in adolescence are polymorphic, nosologically nonspecific, and can lead to diagnoses that are not limited only by the affective pathology. The most common symptoms (irritability, anxiety, mood falls) can act as transdiagnostic phenomena that must be taken into consideration both in the diagnostic study and in further clinical and dynamic follow-ups and treatment.

**Disclosure:**

No significant relationships.

